# Fiber in the Treatment of Dyslipidemia in Pediatric Patients

**DOI:** 10.3390/children12040427

**Published:** 2025-03-28

**Authors:** Maria Elena Capra, Giacomo Biasucci, Elisa Travaglia, Roberta Sodero, Giuseppe Banderali, Cristina Pederiva

**Affiliations:** 1 Pediatrics and Neonatology Unit, Guglielmo da Saliceto Hospital, 29121 Piacenza, Italy; m.capra@ausl.pc.it; 2 Department of Medicine and Surgery, University of Parma, 43125 Parma, Italy; 3 Pediatrics Unit, Clinical Service for Dyslipidemias, Study and Prevention of Atherosclerosis in Childhood, ASST-Santi Paolo e Carlo, 20142 Milan, Italyroberta.sodero@unimi.it (R.S.); cristina.pederiva@asst-santipaolocarlo.it (C.P.)

**Keywords:** fiber, children, dyslipidemia, pediatric

## Abstract

Dietary fiber is present in many food categories (fruits, cereals, vegetables, legumes), and is considered a beneficial component of adult and children’s diets. It is now well-established that dietary intervention is the first line of treatment for childhood dyslipidemia, both as a curative intervention (Familial Hyperchylomicronemia Syndrome, Sitosterolemia) and as an appropriate lifestyle aimed at improving the lipid profile in dyslipidemia, which is associated with early atherosclerosis and an increased risk of cardiovascular disease in adulthood (Familial Hypercholesterolemia, overweight- and obesity-related dyslipidemia). In this paper, we reviewed the main consensus documents to determine the current indications for its use in children and adolescents, and analyzed the few specific papers on the subject in the literature to assess how fiber is currently used in the treatment of pediatric dyslipidemia, what precautions should be taken, and what the main benefits of fiber are on the lipid profile and cardiovascular risk.

## 1. Introduction

### 1.1. Fibers and Healthy Diet

There is a general consensus on the fact that a heart-healthy diet is characterized by a high intake of plant-based foods, such as fruits, vegetables, and whole grains; moderate amounts of protein and dairy products; and small amounts of foods and beverages that are high in fat and sugar [1,2,3]. However, in real life, few populations are close to an optimal diet. A review on the global effect of diet reported that the suboptimal intake of whole grains, fruits, and sodium correlates with half of deaths and two-thirds of disability-adjusted life years related to diet. Nutritional habits, such as the fiber-rich Mediterranean or Nordic diets, have scientifically-proven positive health outcomes, including improvements in blood lipids, blood pressure, and insulin resistance [4].

### 1.2. Fibers and Disease

Several clinical trials and prospective studies have highlighted a correlation between dietary fiber consumption and a reduction in cardiovascular risk [5,6,7,8].

A systematic review and meta-analysis evaluated the risk of cardiovascular disease (CVD) in relation to fiber consumption, finding a 9% reduction in CVD risk (95% Confidence Interval, CI, 6.12%) for every additional 7 g of total fiber consumed per day. The risk ratio for all ten of the included studies was below one, indicating a strong and consistent association. A further analysis of the data showed that the relative risk was greater than one when the total fiber consumption was less than 18 g per day. Increasing fiber intake well beyond 30 g continued to reduce the risk ratio, although with an increasing margin of uncertainty due to the scarcity of data at these high levels of intake. The cumulative estimates for the different types and sources of fiber in the same study showed a lower risk ratio of less than one for soluble, insoluble, cereal, fruit, and vegetable fibers. However, not all risk reductions were statistically significant, likely due to the small number of studies (three to five for each type of fiber), or because not all fibers are equally protective [9].

In a similar review, the same authors reported data on the relationship between fiber consumption and the first episode of stroke, highlighting that total fibers are protective, although it is not clear which types of fibers are decisive in this relationship [10].

They also reported that total fiber intake reduces the risk of type 2 diabetes by about 6% for every increase in 7 g of fiber consumed. Whole foods appear to be particularly protective against type 2 diabetes, with cumulative estimates indicating a risk reduction of up to 32% (95%, CI, 19.42%) for three daily servings of whole grains. Each serving, equivalent to a slice of whole grain bread, reduces the risk by about 10% [11].

Some types of fiber seem to be able to reduce blood pressure in healthy individuals without hypertension. A systematic review of trials investigating the effect of seven different types of fibers showed that diets rich in β-glucans (mean difference of 4 g of β-glucans between control and intervention groups) lowered systolic blood pressure by 2.9 mmHg (95%, CI, 0.9, 4.9) and diastolic blood pressure by 1.5 mmHg (95%, CI, 0.2, 2.7). The effects may be more pronounced in hypertensive individuals [12].

Moreover, β-glucans seem to reduce low-density lipoprotein (LDL) cholesterol in both healthy individuals and those with hypercholesterolemia. A review conducted on trials found an average reduction of 0.15 mmol (95%, CI, 0.09, 0.21) in healthy patients and 0.20 mmol (95%, CI, 0.13, 0.26) in patients with hypercholesterolemia, with an average difference of 3.5 g of β-glucans [12].

### 1.3. Dyslipidemia and Atherosclerosis in Pediatric Patients

Several studies have documented that dyslipidemias represent one of the main cardiovascular risk factors, with effects observable as early as childhood [13,14,15,16]. As summarized in the main specific consensus documents, an early and targeted intervention on risk factors during childhood can significantly improve cardiovascular health throughout the lifecycle and reduce the incidence of cardiovascular events in the population [9,10,11]. In particular, it has been recognized that early and continuous exposure to high levels of LDL cholesterol, a particularly atherogenic fraction of cholesterol, in the bloodstream leads to the early formation of lipid streaks and atherosclerotic plaques on the vascular endothelium, with the possibility of acute myocardial infarction or ischemia, even at a young age. This condition is extreme in individuals affected by homozygous familial hypercholesterolemia (HoFH), where LDL-C levels are very high, and myocardial infarction and cardiovascular death have been reported even in pediatric age [16].

Total and LDL cholesterol levels that define dyslipidemia in children and adolescents indicated by the Expert Panel are slightly more restrictive compared to the normal values for adults and require interpretation by a pediatrician expert in lipid disorders who, if dyslipidemia is detected, will evaluate when to begin treatment by taking into account the family history, dietary habits, pubertal stage, or any inflammatory or hormonal comorbidities that could influence the lipid profile [17,18].

Dyslipidemias can be divided into primary (genetics) and secondary, associated with other conditions, such as metabolic syndrome, chronic drug use, and the presence of endocrine, renal, infectious, inflammatory, or accumulation diseases, as described in Table 1.

Primary forms are mainly due to genetic mutations affecting proteins or enzymes involved in lipid metabolism, resulting in a reduced capacity of the liver to remove atherogenic lipoproteins from the bloodstream, and leading to an accumulation of LDL cholesterol in circulation.

The most common form of genetic hypercholesterolemia is familial hypercholesterolemia (FH), determined by alterations in the LDL receptor, which will be poorly functioning or completely inactive in cases of the homozygous form.

Secondary forms are generally less severe and improve until they disappear after the treatment of the condition that generated them [15,16,17,18].

### 1.4. Manuscript Purpose and Characteristics

The special feature of this review was the analysis of published papers on the use of dietary fiber over a wide time window; we started with the oldest papers describing fiber as a dietary supplement in cardiovascular prevention to the most recent papers on the possible use of fiber in children and adolescents with dyslipidemia.

In this paper, we reviewed the main consensus documents to determine the current indications for its application in children and adolescents, and analyzed the few specific papers in the literature to assess how fiber is currently used in the treatment of pediatric dyslipidemia, what precautions should be taken, and what the main benefits of it are on the lipid profile and cardiovascular health.

## 2. Materials and Methods

The aim of our review was to analyze the role of dietary fibers in the treatment of various forms of dyslipidemia in children and adolescents. The following three steps were taken to conduct this narrative review: conducting the search, looking through abstracts and full texts, and evaluating the findings. To accomplish this, articles from 1980 to 2024 were gathered and chosen from the Pub-Med, EMBASE, Scopus, Science Direct, Web of Science, and Google Scholar databases in order to identify relevant studies that aligned with the review’s progression. The most recent search was conducted in December 2024. The search encompassed double-blind, randomized controlled studies, controlled clinical trials, randomized placebo-controlled trials, and systematic reviews. The following combinations of keywords were used: “fiber” AND “children” OR “pediatric” AND “dyslipidemia” OR “hypercholesterolemia” OR “cardiovascular disease” OR “heart-healthy die” OR “plant-base diet”. We also performed a manual search of the reference lists of the selected studies. Only full papers and journals published in English were included in the search. Following a comprehensive search, the abstracts were examined to ensure they were relevant to the topic at hand. After removing all copies, the remaining articles’ abstracts were examined to make sure they satisfied the review’s inclusion requirements. As a result, the relevant studies concentrating on fiber intake and its relationship with dyslipidemia in pediatric subjects were compiled and analyzed to create an integrated narrative review.

## 3. Fibers: Definition and Characteristic

### 3.1. Definition

Alimentary fiber is a component of plant foods constituted by carbohydrates resistant to the digestive process in the gastrointestinal tract. Alimentary fiber includes non-amylaceous polysaccharides (cellulose, hemicellulose, gum, pectin), oligosaccharides (inulin, fructo-oligosaccharides), and lignin [19]. The main contributors of fiber to the diet are the cell walls of plant tissues, which are supramolecular polymer networks containing variable proportions of cellulose, hemicelluloses, pectic substances, and non-carbohydrate components, such as lignin [20].

Over the past 20 years, the definition of dietary fiber has been the subject of numerous debates. The definition that most recently achieved general consensus dates back to 2008, and was formulated by CODEX Alimentarius, an organization that is part of WHO/FAO in ALINORM 09/32/REP. Therefore, dietary fibers refer to a wide range of plant-based compounds that are not completely digested in the human intestine. Dietary fibers are defined as “polymers of carbohydrates with ten or more monomeric units (MU), which are not hydrolyzed by endogenous enzymes in the human small intestine” and include the following [21,22,23]:

o Resistant oligosaccharides (RO), indigestible, with MU between 3 and 9.
o Non-starch polysaccharides (NSP) derived from fruits, vegetables, cereals, and tubers, whether intrinsic, extracted, chemically, physically, and/or enzymatically modified, or of synthetic origin (MU ≥ 10).
o Resistant starch (RS) with MU ≥ 10.

This subdivision is incorporated in the definition provided by EFSA in 2010 [19]. In addition to the degree of polymerization, other definitions consider the associated substances (e.g., lignin) and health benefits. The European Union in Directive 2008/100 specifies the health benefits derived from the consumption of dietary fibers, including the reduction of intestinal transit time, the increase in stool volume, the fermentability by colonic microflora, the reduction of total blood cholesterol levels, the reduction of postprandial blood glucose levels, and the reduction of blood insulin levels.

A uniform definition is important for harmonizing food labeling and food composition tables at the international level.

Fibers can be found naturally in foods, but they can also be obtained from them through physical, enzymatic, or chemical processes, with demonstrated health benefits based on generally accepted scientific evidence to be considered as dietary fiber [20,21].

### 3.2. Classification

**Figure 1:**
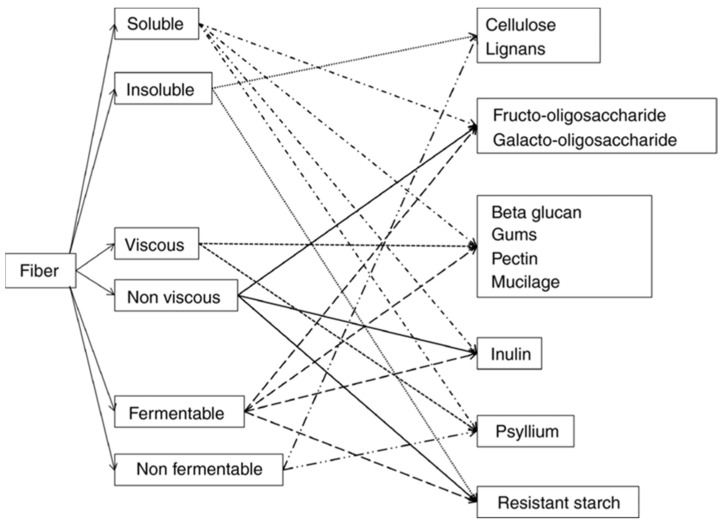
Overlapping properties of fiber by solubility, viscosity, and fermentability (modified from [23]).

From a functional standpoint, dietary fibers can be divided into the following four main classes (Figure 1):

- Insoluble fibers: not soluble in water, with little fermented in the intestine. They may have a possible mechanical laxative effect.
- Non-viscous soluble fibers (inulin, dextrins, oligosaccharides): non-viscous, rapidly fermented, do not increase viscosity, and are completely fermented by the intestinal microbiota. They can exert a prebiotic effect, but without any laxative effect.
- Fermentable viscous soluble fibers (β-glucan, guar gum, pectin): they create a viscous gel in water and increase the viscosity of the chyme, slowing down the absorption of nutrients. They are rapidly fermented in the intestine and lose their laxative effect.
- Non-fermentable viscous soluble fibers (psyllium, multicellulose): they reduce nutrient absorption and, due to their viscosity, can exert a laxative effect.

### 3.3. Mechanism of Action

Fibers influence health through multiple activities that involve different parts of the digestive system; the main mechanisms of action can be summarized as follows [24,25]:

- Colonic function: reduction of transit time, increase in fecal volume, and fermentation in the colon (production of short-chain fatty acids, SCFA). These effects are mainly associated with insoluble fibers such as cellulose, hemicellulose, and psyllium. A diet rich in legumes and whole grains is particularly effective in reducing intestinal transit time.
- Reduction of cholesterol in the blood, with particular mention of β-glucans, which increase viscosity and reduce the reabsorption of bile acids in the small intestine, resulting in a decrease in circulating cholesterol levels.
- Reduction of glucose in the blood and in the small intestine: soluble fibers trap sugars, and the increase in viscosity creates a barrier that slows down glucose absorption, inhibits amylase, and reduces starch digestion, thus improving insulin sensitivity.
- Increased satiety and consequent weight loss: soluble fibers mix with partially digested food in the stomach, slowing its emptying. β-glucans can also stimulate the release of appetite-suppressing substances, such as cholecystokinin, thus increasing the feeling of fullness.

Other mechanisms have also been described, including the improvement of the gut microbiota, hypothesizing that fibers may increase the presence of beneficial bacteria and modulate gene expression, and the reduction of blood pressure [26,27].

### 3.4. Nutritional Fibers’ Sources

Sources of fiber are generally plant-based foods. Cereals mainly provide cellulose and hemicellulose, while fruits and vegetables offer cellulose, hemicellulose, and pectin. Legumes contain hemicellulose, pectin, and resistant starches. In proportion, two-thirds of dietary fiber comes from cereals and only one-third comes from fruits and vegetables. In Table 2, we have reported the average fiber content of the most commonly consumed food items [28].

## 4. Fibers in the Treatment of Dyslipidemia

Fiber, as a positive food [29], has been the subject of numerous scientific studies and is also included in many consensus documents and guidelines for the treatment of lipid disorders.

### 4.1. Key Consensus Pediatric Documents

The WHO report of 2003, extended to the pediatric population, recommends an increase in fiber intake as part of a balanced diet, tailored to both the caloric needs and growth rates of children [30]. The pediatric dietary recommendations of the American Heart Association, published in 2005, indicate a fiber intake of 14 g for every 1000 kcal consumed, with specific values based on age, as follows: 19 g per day for children aged 1–3 years, 25 g per day for those aged 4–8 years, and 29 g/day for girls and 38 g/day for boys aged 14–18 years [31]. Such indications are integrated with those of EFSA, which, in the 2010 document “Scientific Opinion on Dietary Reference Values for Carbohydrates and Dietary Fibre,” provides specific recommendations on fiber intake in children, suggesting an intake of 2 g of fiber per MJ of energy consumed for children over 12 months of age. This level is considered sufficient to ensure normal intestinal function, with indirect positive effects on metabolic and cardiovascular health [19]. The NHLBI (National Heart, Lung, and Blood Institute), in the 2011 guidelines for cholesterol reduction in children, reiterates the importance of fiber intake, recommending an intake of “age + 5 g” per day, emphasizing the importance of a gradual introduction to minimize gastrointestinal discomfort [15,31]. Furthermore, the 2016 guidelines of the European Atherosclerosis Society recognize the crucial role of fibers in the management of dyslipidemias, highlighting that an increase in the consumption of soluble fibers (3–5 g per day) can significantly reduce LDL levels (the “bad” cholesterol), with an average decrease of 5–10% [32,33]. For younger children, the suggested strategy is the “age + 5 g” approach, consistent with recommendations from other international guidelines. Finally, ESPGHAN (the European Society of Paediatric Gastroenterology, Hepatology and Nutrition), in the “Nutritional Recommendations” of 2017, confirms the indications of EFSA, emphasizing that a fiber intake of 2 g per MJ of energy is adequate to support the improvement of the lipid profile in children, but recommending the avoidance of excessive fiber intake, particularly in the form of supplements, to prevent potential nutritional deficiencies [34]. The most recent document was published in 2023 by the WHO [35], and in this document fiber intake recommendations are provided for different pediatric age groups, as you can see in Table 3.

### 4.2. Main Evidence from the Literature

In 1989, the DART study, one of the first RCTs on the role of diet in cardiovascular prevention (secondary prevention), did not show encouraging results on the protective role of fibers. Specifically, 2033 men hospitalized for acute myocardial infarction were analyzed and divided into the following two groups: one with a diet characterized by an increase in polyunsaturated fats over saturated fats and an increase in fiber content, and the other with a free diet. In the two-year follow-up assessment, the incidence of new coronary events was comparable between the two groups, without significant differences [36]. Numerous subsequent studies, however, have increasingly demonstrated the positive effect of fiber on cardiovascular risk. In 2004, a pooled analysis of 10 prospective studies highlighted that an increase of 10 g/day of total fiber was associated with a 14% reduction in coronary events and a 27% decrease in coronary heart disease mortality [37]. In 2009, a study conducted on 769 patients aged between 55–80 years and with risk factors for coronary events such as type 2 diabetes mellitus or having at least three of the following factors—smoking, hypertension, LDL above 160, HDL below 40, body mass index (BMI) > 25, or a family history of early CHD events—showed that an increase in fiber consumption was proportionally associated with a significant reduction in body weight, abdominal circumference, systolic and diastolic blood pressure, fasting blood glucose, and total cholesterol, as well as LDL cholesterol, which are all known risk factors for coronary events [38]. Multiple recent systematic reviews would confirm what has already been highlighted in previous years, namely that significant reductions in LDL cholesterol would be determined, with a high degree of evidence, by soluble or viscous fibers (beta-glucan, glucomannan, oats, barley, pectin, guar gum, psyllium, etc.). The range of total cholesterol reduction would be from 0–18% for oat fibers, 3–17% for psyllium, 5–16% for pectin, and, finally, 4–7% for guar gum [39,40,41,42]. Regarding whole grains, they result in a 2% and 5% reduction in total cholesterol and LDL, respectively [43]. These data may seem to have little relevance, but they are actually significant when considering that a decrease of about 1% in total cholesterol (TC) and LDL levels is associated with a reduction of 2–3% and 1% in cardiovascular risk [32,33]. The positive effect is due to the composition of the cereal itself, and specifically the type of predominant fiber. In this sense, the ones showing the greatest benefits would be oats, barley, and rye, at the expense of wheat. Again, by comparing different classes of whole grains (specifically barley, brown rice, barley, wheat, oats, and the bran of these grains), the authors highlighted the clear superiority in reducing LDL cholesterol and total cholesterol by oat bran first and oats second. On the contrary, barley, brown rice, wheat, and wheat bran would have no impact. The reason for the difference would be due to the prevalence of beta-glucan in oats [44].

## 5. Fibers in Pediatric Patients

The use of fibers as dietary supplements in children and adolescents must take into account the rapid growth, the development of the digestive system, and the changes in taste that occur in the early years of life. During weaning, it is not advisable to exceed 8.4 g/1000 kcal (2 g/MJ) of fiber [45]. It is also necessary to introduce fibers very slowly into the infant’s diet to prevent their intestines, accustomed to a milk-only diet, from reacting negatively, with irritation, colic, or paradoxical constipation. This means starting with foods completely devoid of fiber (e.g., vegetable broth with the addition of pre-cooked gluten-free and non-whole grain flours), and only later checking the tolerance to fiber by gradually introducing vegetable puree and fruit [46,47]. Fibers can also interfere with the absorption of micronutrients like iron, which is vital for physical and cognitive development. A fiber-rich meal, like the one an adult should consume, would tend to fill the child without meeting their immediate nutritional needs, which primarily require energy from fats, proteins, and carbohydrates. Thus, for this reason, in the first year of life, it is better to avoid whole foods, favoring peeled, shelled, or pureed legumes, and offering only small amounts of vegetables [35,47]. In older ages, the main difficulty lies in the general ’novel food rejection’, compared to those that are already known, due to the food’s different color and/or taste, while in school age, especially when offered on the school menu, novel foods are generally well-accepted [47,48].

In the following paragraphs, we will delve into the details of using fibers in the treatment of lipid metabolism disorders.

### 5.1. Heterozygous Familial Hypercholesterolemia

Familial hypercholesterolemia (FH) is one of the most common genetic disorders worldwide. For affected children, drug treatment is currently possible starting from 8–10 years of age, although it is strongly recommended that lifestyle interventions begin at an early age, and that dietary–nutritional treatment plays a fundamental role [16]. In the 2016 consensus document drafted by the EAS (European Atherosclerosis Society), the previous indication [31] on the possible use of fibers as a dietary supplement starting from 6 years of age is reiterated, as the literature’s data have confirmed their safety and efficacy in reducing cholesterol levels, even in children and adolescents [33]. Regarding efficacy, as early as the late 1980s, several studies highlighted the role of fiber as an adjunct food to low-fat and low-cholesterol diets [49]. In this specific case, such combined regimens proved to be more effective than the diet alone. In 1990, Glassman et al., in their study on a cohort of pediatric patients with familial type 2 hypercholesterolemia, demonstrated the usefulness of soluble fibers in reducing the total and LDL cholesterol, as well as the superiority of the combined dietary regimen compared to the step I diet proposed by the American Heart Association [50]. On the contrary, psyllium initially seemed not to have synergistic effects, with the diet in children already subjected to restrictive dietary regimens [51]. Such results were refuted early on [52], and recently, the lipid-lowering role of this fiber was reconfirmed. In particular, a 2015 study conducted by Ribas et al. on 51 pediatric patients aged between 6 and 19 years found, in the intervention group, following the intake of 7 g of psyllium, a reduction of 7.7% in LDL cholesterol and 10.7% in total cholesterol compared to the controls [53]. Regarding glucomannan, this fiber has the peculiar characteristic of reducing triglycerides as well as total and LDL cholesterol, an effect that seems to be determined by its viscosity [54]. These effects have been extensively confirmed in adult patients in a recent meta-analysis conducted by Sood et al., according to which an intake of 1.24 to 15.1 g/day of glucomannan corresponds to a reduction of 15.9 mg/dL of LDL and 11.5 mg/dl of triglycerides compared to the placebo [55]. Similar results have been observed in the pediatric field. Martino et al., in a double-blind study conducted on 40 subjects after 8 weeks of treatment with glucomannan capsules at a dosage of 2 cps/day, report a significant reduction in total cholesterol and LDL levels [56]. In 2013, Guardamagna et al. conducted a double-blind study on 36 children aged between 6 and 15 years with familial hypercholesterolemia; in their study, one group was subjected to a diet rich in glucomannan, and the other to a placebo. After 8 weeks of intake, a significant reduction in total cholesterol, LDL cholesterol, and non-HDL cholesterol was observed compared to the placebo, which is as follows: 5.1%, 7.3%, and 7.2%, respectively [57]. It is interesting to note that both cited studies showed differences between male and female subjects, with greater reductions in total cholesterol (TC) and LDL in the latter. Such results would be attributed to the influence of sex hormones on lipid metabolism. For beta-glucan, its uses in the pediatric field are less represented. In contrast, the lipid-lowering effect is well-documented in the adult population. For example, a 2015 meta-analysis analyzing 70 RCTs with 916 subjects suffering from hypercholesterolemia highlighted a significant reduction in total cholesterol and LDL cholesterol with beta-glucan consumption [58]. Some results in the pediatric field also confirmed those observed for the adult population [59]. Despite the lipid-lowering effects of fibers being widely demonstrated, their role on primary outcomes, such as ischemic heart disease, the number of deaths, and the age of these deaths, is still under study [55,60].

### 5.2. Weight Excess-Related Dyslipidemia

In a somewhat dated review, the authors documented an improvement in the metabolic profile of obese children and adolescents after psyllium supplementation, highlighting a decrease in LDL cholesterol of 2.78–22.8%, and a change in postprandial blood glucose of −12.2–20.2% [61,62]. Several studies published in the following years have confirmed an inverse correlation between fiber consumption and BMI values in young subjects [63,64,65], both in America and Europe [66,67], with a higher risk of developing metabolic syndrome already present in childhood for those with low fiber intake. In 2011, an evaluation was published on a very large population of children and adolescents over a long period of time, which established the importance of consuming less refined foods (whole grain), instead consuming a diet that is richer from a nutritional standpoint and healthier [68,69]. A more detailed study conducted in the Netherlands also highlights a favorable association between fiber intake in childhood and metabolic health in later ages, with a particularly positive effect on the lipid profile; however, the authors describe a better effect for plant-based fibers compared to those derived from cereals [69]. Other authors have focused on a single fiber (glucomannan), trying to highlight its effect on body weight through a systematic review, concluding that, in children and adolescents, the data were not well-defined, and that further studies with a larger sample size and a longer duration were necessary [70,71]. A study from the same period considers a polysaccharide compound (Policaptil Gel Retard), consisting of a mixture of soluble and insoluble fibers on the development of insulin resistance in a cohort of children and adolescents; it is a randomized longitudinal study that highlighted how this preparation (three tablets per day) resulted in a reduction in body weight, the development of metabolic syndrome, and insulin resistance parameters in obese children and adolescents with a family history of type 2 diabetes and obesity [72]. Such a favorable effect was also documented in the study by Fornari et al., which evaluated the effect of this preparation on the insulin response and postprandial lipid profile in a cohort of obese children [73]. In the same year, Fulgoni et al. reported a favorable correlation between fiber consumption and the reduced risk of developing metabolic syndrome at the age of 13–18 years in the National Health and Nutrition Examination Survey (NAHNES) population of children and adolescents [74]; they also documented an improvement in lipid parameters (5–11% reduction in cholesterol levels), blood pressure values (10–23% reduction in diastolic pressure), and the risk of obesity (3–5% reduction). The consumption of minimally refined foods (whole grain) resulted in only a significant reduction in triglycerides’ plasma values (about 52%) that are necessary [74]. In a more recent study, an inverse correlation between fiber intake and metabolically unhealthy obesity (MUO) associated with metabolic syndrome was confirmed in a cohort of adolescents [75]. Finally, as documented in this recent assessment of cardiovascular health among European adolescents (HELENA study), fibers have been confirmed as an integral part of a healthy and sustainable diet, with positive effects on cardiovascular health starting from childhood [76]. Fibers’ effect on CVD reduction in adults and children is summarized in Table 4.

### 5.3. Familial Chylomicronemia Syndrome

Familial Chylomicronemia Syndrome (FCS) is a rare lipid metabolism disorder that is autosomal recessive, characterized by severe hypertriglyceridemia and a risk of pancreatitis. It occurs due to altered functioning of the genes involved in triglyceride metabolism with a high prevalence of pathogenic variants in the gene encoding lipoprotein lipase (LPL), and it is less frequently caused by the malfunctioning of lipase maturation factor 1 (LMF1), glycosylphosphatidylinositol-anchored high-density lipoprotein-binding protein 1 (GPIHBP1), apolipoprotein C2 (ApoC2), and apolipoprotein A5 (ApoA5) on a genetic basis [80,81,82]. In most cases, particularly in pediatric patients, affected individuals benefit significantly from a targeted and specific dietary–nutritional intervention. The diet is characterized by a very restricted lipid intake (fats <15% of total daily calories) and a marked reduction in simple sugars (maximum 10% of total daily calories), while the consumption of low-fat protein foods, such as beans, lentils, chicken (breast), sole, and egg whites, as well as complex carbohydrates, such as brown rice, oats, and quinoa, is recommended [83,84,85,86]. For an adequate caloric intake, supplementation with MCT oil and fat-soluble vitamins is also recommended. Thus, in subjects affected by FCS, soluble fibers can contribute to maintaining a balanced diet with an adequate caloric intake; we did not find specific studies in the literature on this matter, but only a few case reports or case series [87,88,89,90,91].

### 5.4. Sitosterolemia

Sitosterolemia is another rare autosomal recessive disease of lipid metabolism characterized by increased plasma sterols, xanthomatosis, and early atherosclerosis [92,93]. It is caused by increased intestinal absorption and decreased biliary excretion of plant sterols resulting from homozygous or compound heterozygous mutations in either ABCG5 or ABCG8, which encode the sterol efflux transporters, ABCG5 (sterolin1) and ABCG8 (sterolin 2), that pump sterols out to the intestinal lumen or into bile. The clinical and biochemical picture is indistinguishable from homozygous familial hypercholesterolemia (HoFH) by very high LDL and TC plasma values, but unlike these, there is a very marked cholesterol-lowering dietary response [94,95]. However, there are some cases reported in the literature of children who presented with xanthomatosis and extreme hypercholesterolemia after use of a diet rich in plant fiber, both at weaning and at school age. Plant sterols are, in fact, contained in many of the ’healthy’ foods in the cholesterol-lowering diet, and are found in fruits and vegetables, vegetable oil, wheat germ, nuts, seeds, and algae [96,97]. Although it is an extremely rare disease, we should still consider it as a diagnosis when we find ourselves a child with rapid-onset xanthomatosis after the introduction of a diet rich in plant fiber [97,98].

## 6. Conclusive Considerations

In this work, we evaluated dietary fibers in children and adolescents affected by lipid disorders, both on a genetic basis and associated with overweight/obesity. We have delved into the main characteristics of these dietary components, highlighting some peculiar aspects of their use in children during the early years of life. After an overview of the indications contained in the main consensus documents, we reviewed the works present in the literature to examine in more detail their effect on the lipid profile, glucose metabolism, and body weight, finding a generally positive effect both in genetic forms of dyslipidemia (FH and FCS) and in forms associated with overweight/obesity. As expected, studies in children were sporadic and frequently conducted over a short period of time, while the systematic reviews involving both young people and adults were more consistent. Finally, we have highlighted a certain discrepancy in the number of scientific works dedicated to the treatment with fibers of children and adolescents affected by FH, which, after 2015, have practically disappeared, while in the field of the relationship between fibers and overweight/obesity, we found some publications, even in more recent years. This discrepancy could stem from the fact that in 2015 the European consensus document for the diagnosis and treatment of children and adolescents with FH was published, which emphasizes the need for drug treatment to adequately reduce atherogenic cholesterol levels [16].

In conclusion, fibers, especially those found in cereals, seem to have a positive effect on lipid profiles, glucose metabolism parameters, and body weight in children and adolescents. Fiber is part of a healthy and sustainable diet and plays a significant role as a dietary supplement for cardiovascular health from childhood.

## Figures and Tables

**Table 1:** Classification of dyslipidemias, adapted from [17].

Types of Dyslipidemia	Etiology
***Primitive***	
*Monogenic*	- Pathogenic variants of genes LDLR, APOB, PCSK9, LDLRAP1.
*Polygenic*	- Multiple gene variants that increase the risk of dyslipidemia
***Secondary***	
*Unhealthy dietary habits*	- Excess saturated fat - Alcohol abuse
*Endocrinological pathologies*	- Hypothyroidism and hypopituarism - Diabetes mellitus type 1 and 2 - Polycystic ovary syndrome - Lipodystrophy - Acute intermittent porphyria
*Drugs*	- Corticosteroids - Beta-blockers (HDL reduction) - Isotretinoin - Oral contraceptives - Chemotherapy - Antiviral drugs
*Kidneys*	- Nephrotic syndrome - Hemolytic uremic syndrome - Chronic kidney disease
*Hepatic*	- Chronic cholestasis, biliary cirrhosis - Alagille syndrome
*Rheumatologic*	- Lupus - Juvenile rheumatoid arthritis
*Lysosome storage disorder*	- Gaucher, Niemann–Pick, Tay–Sachs
*Infectious*	- Acute and chronic viral infections (especially hepatitis) - HIV
*Others;*	- Kawasaki disease - Anorexia - Klinefelter’s disease - Post-solid organ transplant - Childhood tumors - Pregnancy

**Table 2:** Fiber content in the most commonly eaten food items, adapted from [28].

Cereals and Carbohydrates	Total Fiber/100 g	Nuts and Seeds	Total Fiber/100 g
whole wheat	13–24 g	peanuts	7.6 g
oats	8 g	almonds	7.4 g
bread	7 g	sesame	7.9 g
pasta	4.2 g	sunflower seeds	6.0 g
**Fruit**	**Total Fiber/100 g**	**Vegetables**	**Total Fiber/100 g**
figs	6.9 g	lentils	7.9 g
strawberries	3.8 g	peas	5.6 g
pears	3.1 g	beans	4.9 g
banana	2.6 g	broccoli	2.8 g
oranges	2.4 g	carrots	2.5 g

**Table 3:** Fibers: key consensus pediatric documents.

Source	Age Group	Recommendation
**WHO, 2003 [30]**	All children	Increase fiber intake as part of a balanced diet
**AHA, 2005 [31]**		Recommendation of 14 g of fiber per 1000 kcal consumed
		- 1–3 years: 19 g of fiber per day
		- 4–8 years: 25 g of fiber per day
		- 14–18 years: 29 g of fiber per day for girls, 38 g for boys
**EFSA, 2010 [19,20]**	>12 months	2 g of fiber per MJ of energy consumed
**NHLBL, 2011 [15]**	All children	“Age + 5 g” of fiber per day
**EAS, 2016 [33]**	Children with dyslipidemia	3–5 g of soluble fiber per day (e.g., β-glucans from oats or barley)
	Adolescents	25–30 g of fiber per day, adjusted to energy needs
**ESPGHAN, 2017 [34]**	All children	2 g of fiber per MJ of energy consumed
**WHO, 2023 [35]**	All children	- 2–5 years old, at least 15 g per day - 6–9 years old, at least 21 g per day - 10 years or older, at least 25 g per day

**Table 4:** Fiber effect on CVD risk reduction in adult and pediatric patients.

Type of Fibres	Type of Study	Sample	Dose and Duration of the Intervention	Effects	Reference
General Fibres	RCT (DART Study, 1989)	2033 post-infarction men	5-year follow-up	No significant effect on secondary prevention.	DART Study, *Lancet*, 1989 [36]
	Pooled Analysis	10 prospective studies	Increase of 10 g/day	↓ risk of coronary events by 14%; ↓ CHD mortality by 27%.	Pereira et al., *Arch. Intern. Med.*, 2004 [37]
	Review	15 RCTs with 453 participants	Various doses, duration not specified	No effectiveness for primary outcomes due to lack of data.	Malhotra et al., *Cochrane Database Syst. Rev.* 2014 [60]
Guar Gum	Cross-over Design	28 subjects (11 children and 17 adults): 18 with FH, 10 normal	15 g/day for 8 weeks	Reduction in total cholesterol and LDL in both affected and healthy adults and children.	Zavoral JH et al., *Am. J. Clin. Nutr.*, 1983 [49]
Soluble Fibres	open cross-over study	2–5 year old preschool children	4–10 g/day for 13-weeks	↓ TC: −4%;	Williams, C.L. et al., *Am. J. Dis. Child.*, 1999 [77]
Psyllium	RCT	36 children (3–17 years) with type IIa hypercholesterolemia	≤7 years: 5 g/day; ≥7 years: 10 g/day; duration: 8 ± 1.1 months	↓ TC: −18%; ↓ LDL-C: −23%	Glassman M et al., *AJDC*, 1990 [50]
	RCT	Children and teenagers with high LDL levels after at least 3 months on diet	5–17 years, dose not specified	No statistically or clinically significant differences.	Dennison et al., *J. Pediatr.*, 1993 [51]
	SB-RCT	50 children (2–11 years) with LDL-C ≥110 mg/dL	12-week intervention with cereals containing 3.2 g of psyllium	↓ TC: −9.6%; ↓ LDL-C: −15.7%; ↑ HDL-C: +9.96%	Williams CL et al., *J. Am. Coll. Nutr.*, 1995 [78]
	DB-CO-RCT	32 children (6–18 years) with LDL-C ≥90th percentile	8-week diet: 58 g of cereals with 6.4 g psyllium or placebo	↓ TC: −5%; ↓ LDL-C: −6.8%	Davidson MH et al., *Am. J. Clin. Nutr.*, 1996 [52]
	DB-RCT	51 children (6–19 years) with TC ≥175 mg/dL	6-week intervention with 7 g/day psyllium vs. 7 g/day cellulose (control)	↓ TC: −7.7%; ↓ LDL-C: −10.7%	Ribas SA et al., *Br. J. Nutr.*, 2015 [53]
Beta-Glucan	RCT	29 children (6–14 years)	3 g/day for 4 weeks	↓ LDL-C: -5,3%	Maki, K.C. et al., *Nutr. Res.*, 2003 [59]
	Meta-analysis of RCTs	Adults across multiple trials	3–10 g/day for various durations	↓ LDL-C; ↓ TC; (*p* < 0.00001)	Zhu X. et al., *Nutr. Metab. Cardiovasc. Dis.*, 2015 [58]
Pectin	Non randomized	51 children (4-18 years) with hyperlipidemia, 33 controls	50 mg/Kg/day for 3 months	↓ LDL-C: −17%; ↓ TC: −15%	Sanchez-Bayle, M. et al., *Clin. Pediatr. (Phila)*, 2001 [79]
	Systematic Review and Meta-Analysis	Adults with hypercholesterolemia	every 5 g/day of soluble fibre	↓ LDL-C: −8.28 mg/dL; ↓ TC: −10.82 mg/dL;	Ghavami, A. et al., *Adv. Nutr.*, 2023 [41]
Glucomannan	Review	14 studies	Various doses, duration not specified	Positive effects on TC, LDL, triglycerides, weight, and fasting hyperglycemia, but not on HDL or BP.	Sood N. et al., *Am. J. Clin. Nutr.*, 2008 [55]
	DB-CO-RCT	36 FH children (6–15 years) with TC > 90th percentile	4-week CHILD I diet, 8-week glucomannan or placebo, 4-week washout	↓ TC: −5.1%; ↓ LDL-C: −7.3%	Guardamagna O et al., *Nutrition*, 2013 [57]
	DR-RCT	132 children (3–16 years) with TC ≥170 mg/dL or family CVD history	8-week treatment with 5 neutraceuticals or placebo	GM + CP: ↓ LDL-C: −16%; GM + PC: ↓ LDL-C: −10%	Martino F et al., *Atherosclerosis*, 2013 [56]
konjac glucomannan (KJM)	Meta-analysis of RCTs	12 studies (8 in adult, 4 in children)	A dose of around 3 g/day of KJM significantly reduces LDL cholesterol levels (10%).	↓ LDL-C; ↓ non-HDL-C	Ho et al., *Am. J. Clin. Nutr.*, 2017 [40]

## Abbreviations

The following abbreviations are used in this manuscript:

ApoA5	Apolipoprotein A5
ApoC2	Apolipoprotein C2
BMI	Body Mass Index
CI	Confidence Interval
CVD	Cardiovascular Disease
EFSA	European Food Safety Authority
ESPGHAN	European Society of Paediatric Gastroenterology, Hepatology and Nutrition
FAO	Food and Agricultural Organization
FCS	Familial Chylomicronemia Syndrome
FH	Familial Hypercholesterolemia
GPIHBP1	Glycosylphosphatidylinositol-anchored high-density lipoprotein-binding protein 1
HDL	High-Density Lipoprotein
HoFH	Homozygous Familial Hypercholesterolemia
LDL	Low-Density Lipoprotein
LDLR	Low-Density Lipoprotein Receptor
LDLRAP1	Low-Density Lipoprotein Receptor Adaptor Protein 1
LMF1	Lipase maturation factor 1
LPL	Lipoprotein Lipase
MCT	Middle Chain Triglycerides
MU	Monomeric Units
MUO	Metabolically Unhealthy Obesity
NHANES	National Health and Nutrition Examination Survey
NHLBI	National Heart, Lung, and Blood Institute
NSP	Non-starch Polysaccharides
PCSK9	Proprotein Convertase Subtilisin/Kexin type 9
RS	Resistant Starch
SCFA	Short-Chain Fatty Acids
TC	Total Cholesterol
WHO	World Health Organization
